# Pyridine-2,6-Dithiocarboxylic Acid and Its Metal Complexes: New Inhibitors of New Delhi Metallo β-Lactamase-1

**DOI:** 10.3390/md18060295

**Published:** 2020-06-02

**Authors:** Chris S. Thomas, Doug R. Braun, Jose Luis Olmos, Scott R. Rajski, George N. Phillips, David Andes, Tim S. Bugni

**Affiliations:** 1 Pharmaceutical Sciences Division, University of Wisconsin–Madison, Madison, WI 53705, USA; csthomas4@wisc.edu (C.S.T.); drbraun1@wisc.edu (D.R.B.); scott.rajski@wisc.edu (S.R.R.); 2 Department of Biosciences, Rice University, Houston, TX 77005, USA; olmos@rice.edu (J.L.O.J.); georgep@rice.edu (G.N.P.J.); 3 Department of Chemistry, Rice University, Houston, TX 77005, USA; 4 Department of Medicine, University of Wisconsin School of Medicine and Public Health, Madison, WI 53705, USA; dra@medicine.wisc.edu

**Keywords:** NDM-1 inhibitors, marine-derived *Streptomyces* sp., carbapenem-resistant Enterobacteriaceae, metal chelators

## Abstract

Carbapenem-resistant Enterobacteriaceae continue to threaten human health worldwide with few effective treatment options. New Delhi metallo-β-lactamase (NDM) enzymes are a contributing element that drive resistance to many β-lactam- and carbapenem-based antimicrobials. Many NDM inhibitors are known, yet none are clinically viable. In this study, we present and characterize a new class of NDM-1 inhibitors based on a pyridine-2,6-dithiocarboxylic acid metal complex scaffold. These complexes display varied and unique activity profiles against NDM-1 in kinetic assays and serve to increase the effectiveness of meropenem, an established antibacterial, in assays using clinical Enterobacteriaceae isolates.

## 1. Introduction

Carbapenem-resistant Enterobacteriaceae (CRE) infections are acknowledged by the Centers for Disease Control and Prevention (CDC) and the World Health Organization (WHO) as a growing, high priority health concern [1,2]. In addition to carbapenems, infections of this type harbor resistance mechanisms to numerous antibiotics, leaving few effective treatment options. Studies conducted worldwide have found that the mortality rates for CRE infections often exceed 40% [3,4,5,6,7]. At present, formerly shelved antibiotics such as colistin (polymyxin E) are being reevaluated as treatment options for CRE [8]. Although colistin can offer relief, there is often associated nephrotoxicity with the use of this drug [9], and outbreaks of colistin-resistant CRE have been documented [10,11]. Thus, CRE infections make clear the desperate need for new, safe, and effective treatment options.

Carbapenem resistance in CRE can take several forms, including decreased porin expression [12,13] and upregulated efflux pumps [14,15]. Additionally, β-lactamases capable of hydrolyzing carbapenems (carbapenemases) as well as the other classes of β-lactams have also been a prominent resistance factor found in CRE. Carbapenemases such as *Klebsiella pneumonieae* carbapenemases (KPC) and oxacillinases (OXA) are serine β-lactamases (SBLs, Ambler class A and D, respectively) that employ active site serine residues to hydrolyze the β-lactam pharmacophore [16,17]. Metallo-β-lactamases (MBLS, Ambler class B) also function as carbapenemases, but instead utilize zinc ions in the active site to facilitate lactam hydrolysis. Enzymes in this class include the Verona integron-borne metallo-β-lactamases (VIMs), imipenemases (IMPs), and the New Delhi metallo-β-lactamases (NDMs) [18].

The New Delhi metallo-β-lactamase-1 (NDM-1) was discovered in 2008 from an infection present in a Swedish patient admitted in New Delhi, India [19]. Isolates collected from this patient harbored a plasmid that encoded the new metallo-β-lactamase (NDM-1). Furthermore, the NDM-1-containing plasmid harbored additional resistance elements and an efflux pump that conferred resistance to rifampicin, erythromycin, and gentamycin, thereby compounding the difficulties associated with achieving effective patient treatments. At present, NDM-1 is the most prevalent MBL expressed in CRE infections reported to the CDC in the United States [20]. New and effective treatment options are needed in order to effectively combat clinical infections expressing NDM-1 resistance.

**Figure 1:**
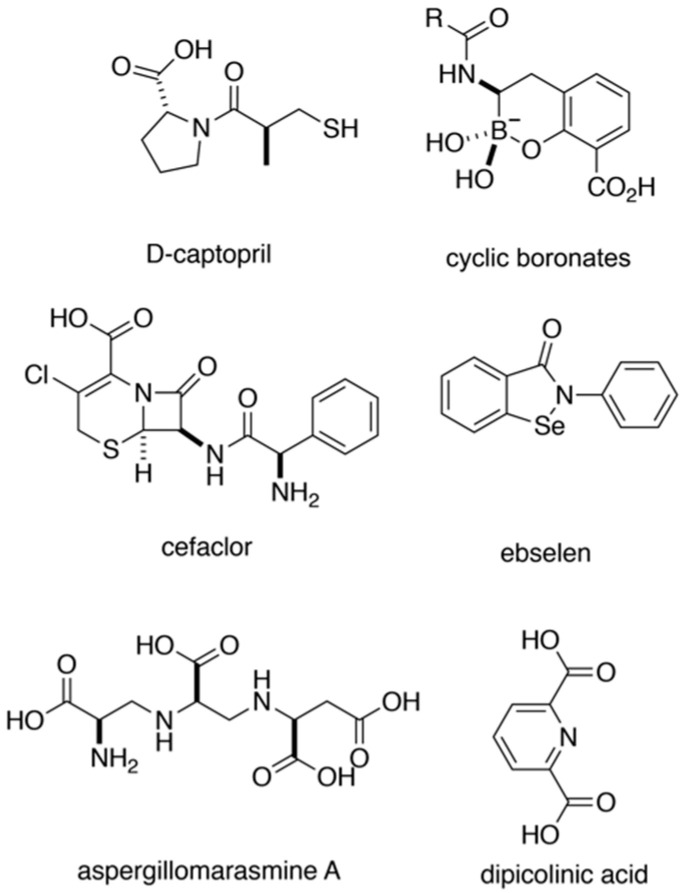
Select New Delhi metallo-β-lactamase-1 (NDM-1) inhibitors from the literature.

The co-administration of β-lactamase inhibitors alongside β-lactam antibiotics has proven to be an effective treatment option that helps to circumvent β-lactamase resistance mechanisms. In particular, recent FDA-approved treatments featuring inhibitors such as tazobactam, avibactam, and vaborbactam continue to demonstrate clinical success in targeting SBL-dependent β-lactam resistance [21]. Many inhibitors that target MBLs, NDM-1 in particular, have also been reported in recent years [22]. These agents display a vast diversity of pharmacophores (Figure 1) and mechanisms of enzyme inhibition. Many of these compounds, such as captopril, feature a thiol group that coordinates with the active site Zn^2+^ in NDM-1, thus interfering with nucleophilic hydroxide production [23,24,25]. In addition, boronic acids, both cyclic (depicted) and noncyclic, inhibit NDM-1 by mimicking the transition state of hydrolyzed β-lactams [26,27] and have shown recent promise, currently in late-stage clinical development [28]. Metal chelators such as dipicolinic acid and the natural product aspergillomarasmine A knock out NDM-1 activity by stripping the active site of Zn^2+^ [29,30]. The covalent inhibition of NDM-1 has also been documented in a few cases with compounds such as ebselen and cefaclor, compounds that target cysteine and/or lysine residues in the active site [31,32]. Despite these advances and expanding insights into the mechanisms of NDM-1 inhibition, there remain no FDA-approved inhibitors of NDM-1. The absence of approved agents necessitates continued investigations for new sources of drug leads.

In the marine environment, iron is a growth-limiting nutrient owing to its essential role in numerous microbiological functions and sub-nanomolar concentrations in oceanic surface waters [33]. Marine microorganisms sequester iron predominantly through chelating compounds termed siderophores. The structural diversity of marine siderophores is vast and has been reviewed recently [34]. However, despite this breadth of study, new metal-chelating compounds continue to be discovered from the marine environment [35,36]. While many siderophores have been studied for their propensity to bind iron, studies with other metals are limited [37,38]. Additionally, the natural proclivity for the microbial uptake of these metal-bound species favors their potential development as antibiotic drug leads [39]. Taken together, the marine environment represents a unique niche for the discovery of new metal-binding inactivators of NDM-1.

During mass spectrometry (MS)- and NMR-guided investigations of marine-derived actinomycete extracts for the discovery of new bioactive natural products, we found iron-bound pyridine-2,6-dithiocarboxylic acid (PDTC_2_-Fe) from the culture extract of marine *Streptomyces* strain WMMB-314. PDTC is a known metal chelator that was originally discovered from a then-unidentified *Pseudomonas* species. Following the addition of Fe^3+^ to the *Pseudomonas* culture medium, PDTC bound to iron (PDTC_2_-Fe) was isolated [40]. PDTC has since been identified and studied in several *Pseudomonas* spp. and has been associated with the transport of iron and zinc in these organisms [41,42,43]. Previous studies showed that the PDTC ligand alone effectively inhibited the growth of non-*pseudomonas* bacterial spp. but lost antimicrobial activity in the presence of certain transition metals such as iron or cobalt. Notably, zinc enhanced the antimicrobial activity of PDTC [44]. In a separate study, the metal binding and antimicrobial properties of PDTC with a wider array of transition metals were investigated, demonstrating that many other PDTC–metal complexes enhanced antimicrobial activity compared to PDTC alone [45]. 

Given the structural relatedness between PDTC and the NDM-1 inhibitor dipicolinic acid, we hypothesized that PDTC would also demonstrate similar inhibitory behavior towards this enzyme. In fact, Roll and co-workers found that several analogs of dipicolinic acid, including PDTC, inhibited a variety of bacterial MBLs and mammalian metallo-enzymes that contain zinc [46]. In this investigation, we examined PDTC activity against NDM-1-harboring CRE pathogens in conjugation with meropenem, and also against NDM-1 in kinetic assays. Importantly, the unique activity observed in the kinetic assays encouraged us to examine several metal-bound PDTC complexes as potential inhibitors of NDM-1 as well. PDTC–metal complexes displayed different NDM-1 inhibition activity depending on the metal bound. Additionally, complexes were also able to rescue meropenem’s efficacy against clinical NDM-1 CRE isolates.

## 2. Results

### 2.1. Identification, Synthesis and Evaluation of PDTC against Clinical CRE Isolates with Meropenem

**Figure 2:**
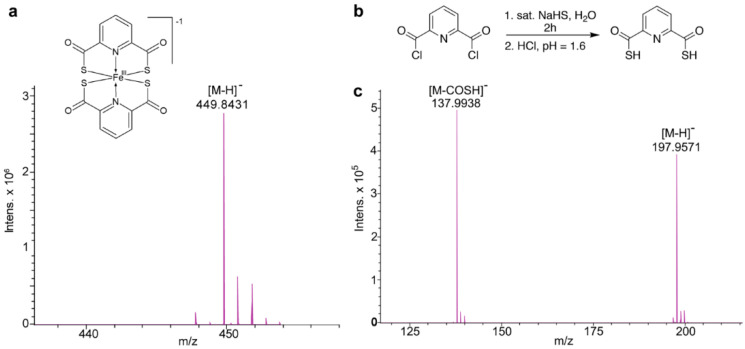
(**a**) Structure of iron-bound pyridine-2,6-dithiocarboxylic acid (PDTC_2_-Fe) assembly and accompanying negative mode ESI MS analysis. (**b**) Synthesis of PDTC. (**c**) Mass spectrum of PDTC collected using negative mode ESI.

We isolated PDTC_2_-Fe from an extract of marine-derived *Streptomyces* sp. (strain WMMB-314). A chloroform partition from a 10 L culture extract was fractionated using Sephadex LH-20, and subsequent purification via HPLC yielded a black-brown solid. Mass spectrometry revealed a molecular ion with a unique isotope pattern suggesting the presence of iron (ESI(−) *m/z* 449.8431 [M − H]^−^, Figure 2a), and this was consistent with the broad resonances observed in the NMR spectrum. Using the accurate mass measurements for an Antibase [47] database search, we confirmed the identity as PDTC_2_-Fe.

As previously discussed, we hypothesized that metal-free PDTC would be sufficient to inactivate NDM-1. Specifically, we reasoned that PDTC could extract Zn^2+^ from the NDM-1 active site in a fashion similar to that established for dipicolinic acid (Figure 1), which features carboxylic acids instead of the thiocarboxylic acids on PDTC [30]. Using conventional bench-top chemistry and simple reagents/conditions, we readily generated ample amounts of PDTC to support initial NDM-1 inhibition studies (Figure 2 and Figure S1).

**Figure 3:**
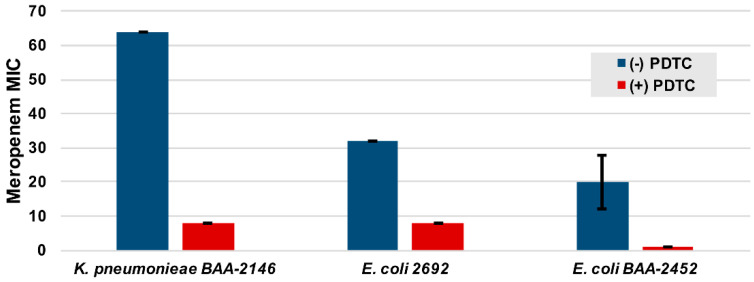
Meropenem MICs in the presence and absence of the PDTC ligand when incubated with clinical pathogens expressing NDM-1. The PDTC concentrations in the relevant samples were 0.25× the PDTC MIC (100 μM for all isolates). All the data represent an average of four replicates with standard deviation error bars.

We began by testing PDTC against three clinical Enterobacteriaceae isolates harboring the NDM-1 gene in the presence of the carbapenem antibiotic and the NDM-1 substrate meropenem. Since PDTC has documented antimicrobial activity, we first collected minimum inhibitory concentration (MIC) data for PDTC against each of the clinical isolates (Table S1) [44]. Meropenem MICs were subsequently collected against these isolates, both in the absence and presence of PDTC at 0.25× its individual MIC. PDTC rescued the antimicrobial activity of meropenem, reducing its MIC against each strain by 4-fold or greater (Figure 3), providing some support for our hypothesis.

### 2.2. Kinetic Evaluation of PDTC against Recombinant NDM-1

To confirm that the reduced meropenem MICs were the result of NDM-1 inhibition by PDTC, we conducted a kinetic analysis of PDTC with isolated enzyme. Recombinant, soluble NDM-1 was expressed and purified from *E. coli* BL21-DE3 cells [48]. CENTA ((6*R*,7*R*)-3-[(3-carboxy-4-nitrophenyl)sulfanylmethyl]-8-oxo-7-[(2-thiophen-2-ylacetyl)amino]-5-thia-1-azabicyclo [4.2.0]-oct-2-ene-2-carboxylic acid), a colorimetric cephalosporin substrate for NDM-1, was used to monitor the reaction rate of the NDM-1 kinetic assays. Zn^2+^ was also supplied to the enzyme in excess (10^5^x or greater) prior to the assay to support a saturated metallo-enzyme binding site. Using this approach, we first investigated the NDM-1 hydrolysis of CENTA in the presence of PDTC.

**Figure 4:**
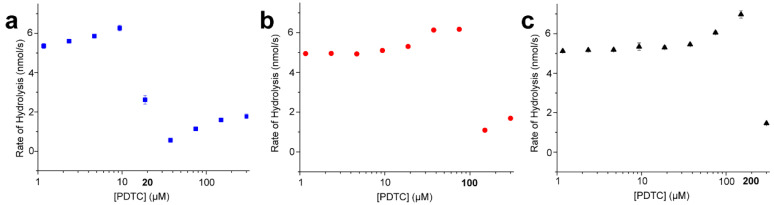
Initial rates of CENTA hydrolysis by NDM-1 vs. PDTC concentration with (**a**) 10 μM ZnSO_4_, (**b**) 50 μM ZnSO_4_ or (**c**) 100 μM ZnSO_4_ supplementing the reaction buffer. In each case, NDM-1 activity was reduced as the PDTC concentration exceeded two equivalents of the ZnSO_4_ in the reaction, denoted by bold type on the *x*-axis.

Two key findings came out of this study. First, two equivalents of PDTC relative to the amount of excess Zn^2+^ included in the NDM-1 assay led to the elimination of NDM-1 activity, followed by some hydrolysis of CENTA due to high concentrations of PDTC (Figure S2). The incubation of PDTC with ZnSO_4_, the metal salt used for the assay, afforded a 2:1 PDTC/Zn complex (PDTC_2_-Zn) as revealed by MS analysis (ESI(−) *m/z* 228.90 [M − 2H]^2−^, molecular formula C_14_H_6_O_4_S_4_N_2_Zn), along with several 1:1 complexes containing different anionic ligands (Figure S3). When the Zn^2+^ concentrations in the colorimetric CENTA cleavage assay were increased, more PDTC was required to eliminate activity, maintaining a stoichiometric 2:1 PDTC/Zn ratio for NDM-1 inactivation. These data clearly support the notion that PDTC inactivates NDM-1 by sequestering Zn^2+^ from the active site (Figure 4).

The second main finding from these kinetic assays was that PDTC concentrations lower than twice the amount of Zn^2+^ present (i.e., fewer than two equivalents of PDTC relative to Zn^2+^) led to slightly enhanced rates of NDM-1-catalyzed hydrolysis. In other words, NDM-1 hydrolyzed CENTA at a faster rate than without any PDTC present (Figure 4). Importantly, although NDM-1 was found to be most active between 20 and 40 μM ZnSO_4_ (Figure S4), there was a clear enhancement of NDM-1 activity with PDTC at 10 μM ZnSO_4_ (Figure 4a), suggesting that PDTC does not simply tune the ZnSO_4_ concentration for optimal NDM-1 catalytic efficiency. Additionally, experiments with EDTA could not reproduce the rate enhancement observed with sub-stoichiometric concentrations of PDTC (Figure S5). Taken together, the two findings suggest that a PDTC-Zn complex formed in solution during the assay enhanced NDM-1 activity, presumably via allosteric associations. Once all the excess Zn^2+^ was sequestered by exogenous PDTC, NDM-1 activity was abolished. We posit that en route to the generation of the PDTC_2_-Zn complex, intermediate 1:1 complexes transiently increased NDM-1 activity until all the available Zn^2+^ was sequestered.

### 2.3. Synthesis and Kinetic Evaluation of PDTC Complexes against Recombinant NDM-1

**Figure 5:**
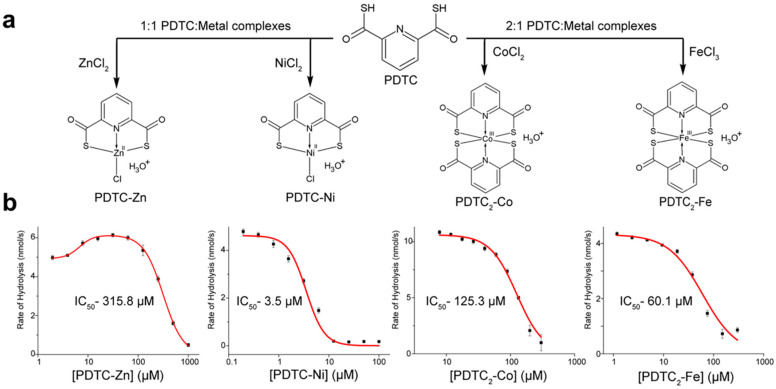
(**a**) General synthesis scheme and stoichiometry for the PDTC–metal complexes as confirmed by mass spectrometry (Figure S6). (**b**) Dose response curves for each of the PDTC–metal complexes against the initial NDM-1 hydrolysis rates of CENTA. PDTC-Zn data were fit using a bi-phasic model to afford a better fit.

Intrigued by the rate enhancement activity observed in the kinetic assay, we next sought to study the effect of individual PDTC metal complexes on NDM-1 kinetics. In particular, we sought to determine whether or not a PDTC-Zn complex prepared in situ before CENTA hydrolysis reactions could enhance NDM-1 activity. We also wished to study how other PDTC complexes might impact NDM-1 activity. To answer those questions, chloride salts of iron, cobalt, nickel and zinc were all combined with the PDTC ligand and characterized via mass spectrometry (Figure S6). The iron and cobalt chloride salts formed 2:1 PDTC/metal complexes, whereas the exposure of PDTC to nickel chloride resulted in a 1:1 complex. Interestingly, although a mixture of 2:1 and 1:1 PDTC-Zn complexes were generated using ZnSO_4_ as a starting material for complex synthesis, the use of ZnCl_2_ as the metal source gave rise only to 1:1 complexes. Accordingly, we generated PDTC complexes using ZnCl_2_ with a 1:1 stoichiometry (Figure 5a). 

Each complex was independently titrated into a kinetic assay at different concentrations, and the initial rates of CENTA hydrolysis were determined (Figure 5b). As anticipated, the 1:1 PDTC-Zn complex enhanced the rate of CENTA hydrolysis by NDM-1 by as much as 34%, before beginning to inhibit the enzyme at concentrations around 100 μM. By contrast, the other 1:1 metal complex, PDTC-Ni, potently inhibited NDM-1. For the 2:1 complexes, PDTC_2_-Fe was more inhibitory towards NDM-1 than PDTC_2_-Co; both complexes failed to enhance NDM-1 activity. 

### 2.4. Evaluation of PDTC Complexes against Clincal CRE Isolates with Meropenem

Having established NDM-1 inhibitory trends with the PDTC–metal complexes, each complex was then tested in combination with meropenem using the previously studied clinical isolates (Figure 3). We hypothesized that the PDTC complexes might also enhance the susceptibility of these isolates as had been observed with metal-free PDTC. We also proposed, based on earlier findings, that the inclusion of PDTC-Zn may counteractively increase meropenem MICs, given this complex’s ability to slightly improve the NDM-1 used for in vitro studies. As before, MICs were first determined for each PDTC complex against each of the three clinical Enterobacteriaceae strains (Table S1). The meropenem MICs were then also determined in the presence of the different metal–PDTC complexes where again, each metal agent concentration was set to 0.25× its MIC (in the absence of meropenem). Table 1 lists the meropenem MICs in the presence and absence of each metal–PDTC complex, as well as the previous data obtained with the free PDTC ligand. 

In general, PDTC–metal complexes rescued meropenem activity, at times reducing the MIC by as much as 10-fold. The PDTC ligand alone demonstrated the greatest enhancement of meropenem activity. In contrast to expectations, we found no evidence that the PDTC-Zn complex enhanced meropenem resistance, although, in two cases, the meropenem MIC was not reduced at all. Interestingly, the meropenem MICs with PDTC_2_-Fe were comparable to those with PDTC-Ni against Enterobacteriaceae despite the ~10-fold difference in kinetic efficacy in favor of PDTC-Ni. Overall, our studies demonstrate that, in addition to the PDTC ligand, metal-bound PDTC complexes are able to interfere with NDM-1 activity.

## 3. Discussion

In summary, a new class of NDM-1 inhibitors has been characterized. PDTC rescued the efficacy of the β-lactam antibiotic meropenem against NDM-1-harboring clinical isolates and furthermore demonstrated NDM-1 inhibition in kinetic assays. Secondary studies with PDTC–metal complexes in kinetic investigations revealed varying inhibitory activities against NDM-1. PDTC-Ni showed the most potent inhibitory activity against NDM-1 kinetically, whereas PDTC_2_-Fe and PDTC_2_-Co showed progressively reduced inhibitory activities. Interestingly, PDTC-Zn enhanced NDM-1 activity, we suspect by enabling allosteric associations that translated into slightly improved NDM-1 function. PDTC appeared to provide optimal reductions in meropenem MIC values against clinical isolates expressing NDM-1, although meropenem MIC values were reduced in the presence of PDTC–metal complexes as well.

During our investigations of transition metal drugs and metal complexes for new inhibitors of NDM-1 and other MBLs, Chen and colleagues identified cisplatin and Pd complexes as potent inhibitors of MBL activity [49]. From their data, they posit that the metal or ligand-bound metal species replaces one of the zinc ions in the active site. In this way, metal chelators with bound metals appear not to strip NDM-1 of active site Zn^2+^ so much as to simply swap out the vital metal–amino acid associations necessary for optimal enzyme function. We have yet to conduct analogous investigations with our metal-bound PDTC species to determine if similar interactions are taking place. Nevertheless, both of our studies corroborate the notion that metal-bound chelator compounds are capable of reducing NDM-1 enzymatic activity; clinically speaking, it is this realization that bears the greatest significance. 

## 4. Materials and Methods 

### 4.1. Reagents, Chemicals and Instrumentation

Clinical Enterobacteriaceae isolates containing NDM-1 were provided by Dr. David Andes’ lab at the University of Wisconsin–Madison. All other solvents, reagents and media components were obtained from commercial sources. NMR datasets were collected with a Varian 500 MHz instrument. Mass spectrometry data were collected on a Bruker MaXis 4G qTof. Protein quantification was conducted using a Thermo Scientific Nanodrop 2000c Spectrophotometer unless otherwise noted. Kinetic assays were read in Costar® polystyrene 96-well plates using a BioTek H1 plate reader.

### 4.2. Purification and Identification of PDTC_2_-Fe

Pyridine-2,6-dithiocarboxylic acid bound to iron (PDTC_2_-Fe) was purified from 10 × 1 L cultures of *Streptomyces* strain WMMB 314 in ASW-A medium (20 g of soluble starch, 10 g of glucose, 5 g of peptone, 5 g of yeast extract and 5 g of CaCO_3_ per 1 L ASW) supplemented with 50 µM FeCl_3_ and 70 g of HP-20 resin. Following 1 week of growth at 28 °C with 200 rpm shaking, cells and resin were collected in Miracloth, washed with DI water, and extracted in acetone. The crude extract (27.29 g) was liquid–liquid partitioned between 90% MeOH in water and hexanes (480 mg). Water was added to the aqueous fraction to make the solution 70% MeOH, and then the solution was partitioned with CHCl_3_ (2.8 g). Then, 800 mg of the CHCl_3_ partition was dissolved in 1:1 MeOH/CHCl_3_ and fractioned using Sephadex LH-20 into four fractions. Fraction 3 contained PDTC_2_-Fe, and 19 mg was injected on a Phenomenex C18 Gemini preparatory HPLC column (250 × 30 mm, 5 µm) using a gradient of MeOH and 10 mM ammonium acetate in water: 0–0.7 min with 40% MeOH, a linear gradient at 0.7–35.2 min to 100% MeOH, and a hold at 100% MeOH at 35.2–42 min. Fractions were collected every 0.5 min beginning at 5 min; Fractions 14–18 contained PDTC_2_-Fe (8.1 mg). Direct infusion MS data collected in (−)-ESI mode revealed a single signal with a unique isotope pattern denoting the presence of iron (*m*/*z* [M − H] = 449.8431). The MS data were parsed on the natural product database Antibase [47], which suggested PDTC_2_-Fe as the metabolite. The compound was confirmed through later synthesis and subsequent MS analysis of PDTC_2_-Fe.

### 4.3. PDTC Synthesis

PDTC was synthesized using methodology adapted from previous work by Chatterjee and Crans [50]. Briefly, 417 mg (2.04 mmol) of 2,6-pyridine dicarbonyl dichloride was added to 10 mL of saturated NaHS in water and stirred for 2 h. HCl [aq, 2N] was then added dropwise to reduce the pH to 1.6, and the solution was stirred for an additional 50 min. Pyridine-2,6-dithiocarboxylic acid precipitated and was collected by partition with dichloromethane (DCM) and dried under a gentle stream of argon to afford 232 mg of a white solid. The sample was loaded onto a 5 g silica gel column (1.75 cm diameter). An unidentified product was washed away with 1:9 dichloromethane (DCM)/hexanes. Elution with 100% CH_2_Cl_2_ then afforded PDTC as a white solid. Notably, a brown color formed on the column as PDTC eluted, likely signaling the formation of PDTC_2_-Fe; silica is a known iron source. Despite this concern, the suspected PDTC_2_-Fe complex was retained on the silica gel column. PDTC was then dried and stored under argon at −20 °C until use. The final yield of pure PDTC was 203 mg (1.09 mmol, 49.9%). ^1^H NMR (CD_2_Cl_2_) δ 8.19 (AB_2_ d, 2H, J = 7.8 Hz), δ 8.11 (AB_2_ t, 1H, J = 7.8 Hz), δ 5.72 (br, 2H). The AB_2_ (Pople nomenclature) exhibited second order effects. The chemical shifts and the coupling constant in this system were calculated and confirmed using the WinDNMR simulation software [51].

### 4.4. Mass Spectrometry of PDTC Complexes

PDTC was dissolved in Ar-purged *N*,*N*-dimethyl formamide (DMF) and then combined with solutions of ZnCl_2_, FeCl_3_, NiCl_2_ and CoCl_2_ to form PDTC complexes at 100 μM. For Co and Fe complexes, a 2:1 PDTC/metal salt stoichiometry was used; 1:1, for Ni and Zn complexes. PDTC was also prepared at 100 μM. These solutions were further diluted to 1 μM in CH_3_CN for direct infusion. The complexes were analyzed by ESI in negative mode at a flow rate of 180 μL/h, a capillary voltage of 2500V, a nebulizer at 0.3 bar, dry gas at 4 L/min, dry temp at 180 °C, and a mass range of 50–1700 m/z.

PDTC was also analyzed in the presence of ZnSO_4_ using MS. PDTC ligand (50 μM) was combined with 1 mM ZnSO_4_ in LCMS-grade H_2_O and reacted at room temperature for 1.5 h. The sample was diluted 1:10 in LCMS-grade acetonitrile for direct infusion under conditions identical to those noted above.

### 4.5. NDM-1 Purification

The pNic28-Bsa4 plasmid containing the NDM-1 construct (pNic-NDM1) was generated as follows. The coding DNA sequence for NDM-1 was ordered from Integrated DNA Technologies (Coralville, IA) as a double-stranded DNA fragment comprising residues 29–270, excluding the signaling peptide coding region, residues 1–28. This DNA fragment was then cloned into pNIC28-Bsa4 via Gibson Assembly using reagents from New England Biolabs (Ipswich, MA, USA). This necessitated the generation of overlapping regions through PCR amplification using the primers provided in Table 2 (below), which were also purchased from IDT. These primers were designed to incorporate the NDM-1 coding DNA sequence just after the TEV protease sequence site on the vector backbone. The pNIC28-Bsa4 empty backbone was obtained from Addgene, plasmid #26103 (Cambridge, MA), where it was deposited by Opher Gileadi on behalf of the Structural Genomics Consortium. Details of the pNic-NDM1 plasmid are available for viewing at the following link: https://benchling.com/s/seq-ZvaWkjSHai7TZYdm8xyH/edit. 

BL21-(DE3) *E. coli* cells were transformed with pNic-NDM1 and plated onto kanamycin (34 μg/mL) selective media. An overnight starter culture of pNic-NDM1 *E. coli* was diluted into a 500 mL culture of LB media. After 4 h (OD_600_ = 0.741), the culture was induced with 0.1 mM isopropyl-β-d-thiogalactopyranoside. The culture was shaken at room temperature for just over 24 h to induce expression. The cells were pelleted and frozen until use; the cell pellets (wet) weighed approx. 4 g. 

Cells (2 g) were combined with 10 mL of Bug Buster® HT Protein Extraction Reagent (EMD Millipore) cell lysis reagent and incubated with gentle mixing at room temperature for 20 min. Cell debris was pelleted and the 10 mL supernatant was combined with 10 mL of wash buffer (50 mM NaH_2_PO_4_, 300 mM NaCl, pH 7.4) and loaded in multiple batches onto a single HisPur™ Cobalt Spin Column (1 mL, Thermo Scientific). Each batch was gently mixed for 30 min before centrifugation for 2 min at 700 g. The column was then washed with wash buffer four times (2 mL at 700 g). The His-tagged NDM-1 was then eluted with elution buffer (50 mM NaH_2_PO_4_, 300 mM NaCl, 150 mM imidazole pH 7.4) four times with 2 mL at 700 g. The elution fraction was spin-concentrated (Amicon® Ultra-15 10K Centrifugal Filter, EMD Millipore) into TEV reaction buffer (20 mM Tris, 0.01 mM ZnCl_2_, 200 mM NaCl, 5 mM sodium citrate, 5 mM 2-mercaptoehtanol, pH 7.4) for TEV cleavage of the His-tag. 

His-tagged NDM-1 was quantified using a Coomassie (Bradford) Protein Assay kit (Thermo Scientific). The His-tagged TEV protease was generated as previously described [52].

Protease was added at 1/20 the mass of NDM-1 and incubated with shaking at room temperature overnight (~18 h). The reaction mixture containing His-tagged TEV, cleaved NDM-1 and the removed NDM-1 His-tags was spin concentrated into wash buffer with 10% glycerol. The mixture was loaded onto a HisPur™ spin column and gently mixed for 30 min. The column was spun for 2 min at 700 g to collect the unbound NDM-1 and was washed three times with wash buffer containing 10% glycerol to collect any remaining NDM-1. The column was also separately washed with elution buffer to remove bound proteins. Fractions of pure NDM-1 were combined, spin concentrated and quantified using a Nanodrop and the calculated extinction coefficient for NDM-1 based on its amino acid sequence [53]. The calculated yield of NDM-1 was 11 mg per liter of culture. Aliquots were frozen at −80 °C until further use. 

### 4.6. Kinetic Data Collection 

PDTC was dissolved in Ar-purged DMF and combined with solutions of ZnCl_2_, FeCl_3_, NiCl_2_ and CoCl_2_ to make concentrated stock solutions of 20 mM in 1:1 water/DMF solutions. The solutions were diluted in assay buffer (HEPES 50 mM, 1 μg/mL of bovine serum albumin (BSA), 0.01% Triton X-100, pH 7.2) to the final testing concentrations through serial dilutions in 96-well flat-bottom plates. NDM-1 was diluted from frozen stocks to a final testing concentration of 100 pM in assay buffer supplemented with 10 μM ZnSO_4_ and aliquoted into the 96 well plates. A larger concentration of NDM-1 (250 pM) was used for PDTC_2_-Co to better establish an initial rate above the noise generated at high concentrations of the complex. CENTA was prepared and added at a final concentration of 50 μM. The plates were analyzed using a kinetic method monitoring the hydrolysis of CENTA at 410 nm over 10 minutes. The initial rates were calculated and used in the generation of dose–response plots in the Origin 9.1 software. The model used was dose–response, under non-linear curve fitting. PDTC-Zn was fit using a bi-phasic dose–response model, in order to better fit the curve and calculate the IC_50_ more accurately. The A1 (bottom) asymptote was held constant at zero for all models to afford a better fit. The kinetic data recorded in the Supporting Information were collected using the same assay buffer as above, with the concentrations of ZnSO_4_ noted. Working solutions of EDTA were prepared from a 0.5 M stock solution (pH = 8).

### 4.7. MIC Determinations

The minimum inhibitory concentrations for PDTC, the PDTC–metal complexes, and the metal salts were determined against three different Enterobacteriaceae isolates that house NDM-1 resistance genes (*E. coli* 2692, *E. coli* BAA-2452 and *K. pneumonieae* BAA-2146) following the Clinical Laboratory Standards Institute (CLSI) guidelines [54]. For the meropenem MICs collected in the presence of PDTC or PDTC metal complexes, methods were adapted from the CSLI guidelines for determining MICs. PDTC and complexes were prepared in DMF as described above and subsequently diluted in cation-adjusted Mueller Hinton Broth (MHB) to the final concentrations shown in the footnote of Table 1. Medium solutions containing these compounds were aliquoted into 96-well plates. Meropenem was added and serially diluted from Columns 12 to 3 in the 96-well plates. The strains were grown overnight in LB media and then diluted to approximately 5 × 10^5^ CFU/mL. The MICs were determined between Columns 3 to 12; Column 2 was a growth control, and Column 1 was a medium control. The DMF was kept at or below 2% in screening conditions. The meropenem MICs collected with metal salts were collected in a similar manner; the metal salts were initially dissolved in DI H_2_O instead of DMF.

## Figures and Tables

**Table 1:** Meropenem MIC (μg/mL) data with each PDTC complex and PDTC alone at the concentrations listed. The MICs shown are average of four replicates.

Complex	*E. coli* 2692	*E. coli* BAA-2452	*K. pneumonieae* BAA-2146
No PDTC or Complex	64 ^a^	20 ^a^	64 ^a^
PDTC_2_-Fe	16 ^a^	2 ^a^	20 ^a^
PDTC_2_-Co	28 ^a^	4 ^a^	32 ^a^
PDTC-Ni	16 ^a^	2 ^a^	32 ^a^
PDTC-Zn	64 ^c^	12 ^c^	64 ^b^
PDTC (no metal)	8 ^b^	1 ^b^	8 ^b^

PDTC complex or PDTC screened at 0.25× its previously determined MIC―^a^ 400 μM, ^b^ 100 μM, ^c^ 50 μM. The metal salts used to generate these complexes were also studied against these organisms in an identical manner (Tables S2 and S3).

**Table 2:** pNIC-NDM1 cloning primers.

Primer	Sequence
pNIC-Fwd	CGCATGGCCGATAAGTTACGTTAAacggtctccagtaaaggt
pNIC-Rev	CCCGATAGTTGGACGAATTTCACCggattggaagtacaggttctc
NDM1-Fwd	accgagaacctgtacttccaatccGGTGAAATTCGTCCAACTATC
NDM1-Rev	gtatccacctttactggagaccgtTTAACGTAACTTATCGGCCA

## Supplementary Materials

The following are available online at https://www.mdpi.com/1660-3397/18/6/295/s1, Figure S1: ^1^H NMR spectrum of synthetic PDTC, Figure S2: Rate of CENTA hydrolysis in the presence of PDTC alone, Figure S3: Mass spectra of PDTC with ZnSO_4_, Figure S4: Rate of CENTA hydrolysis by NDM-1 in assay buffer with ZnSO_4_, Figure S5: Rate of CENTA hydrolysis by NDM-1 in ZnSO_4_ with EDTA, Figure S6: Mass spectra of PDTC complexes collected in ESI (−) mode, Table S1: PDTC complex and PDTC MIC data with CRE isolates, Table S2: Metal salt MIC data with CRE isolates, Table S3: Meropenem MIC data with metal salts.
