# Supplementary material for: Pyridine-2,6-Dithiocarboxylic Acid and Its Metal Complexes: New Inhibitors of New Delhi Metallo β-Lactamase-1
Source: Mar Drugs. 2020 Jun 2;18(6):295. doi: 10.3390/md18060295 (PMC7374359; doi:10.3390/md18060295)
Supplement: Supplementary file 1 [file marinedrugs-18-00295-s001.pdf]

Supporting Information

Pyridine-2,6-dithiocarboxylic Acid and Its Metal Complexes: New Inhibitors of New Delhi Metallo β-lactamase-1

Chris S. Thomas,[a] Doug R. Braun,[a] Jose Luis Olmos, Jr.,[b] Scott R. Rajski,[a] George N. Phillips, Jr.,[b,c] David Andes,[d] and Tim S. Bugni[a,*]

[a]Pharmaceutical Sciences Division, School of Pharmacy University of Wisconsin-Madison, Madison, Wisconsin 53705, USA
[b]Department of Biosciences, Rice University, Houston, Texas 77005, USA
[c]Department of Chemistry, Rice University, Houston, Texas 77005, USA
[d]Department of Medicine, University of Wisconsin School of Medicine and Public Health, Madison, Wisconsin 53705, USA

Table of Contents

Item Name	Contents/Description	Page
**Figure S1**	^{1}H NMR spectrum and integration of synthetic PDTC	S3
**Figure S2**	Rate of CENTA hydrolysis in presence of PDTC alone	S3
**Figure S3**	Mass Spectra of the PDTC with $ZnSO_4$	S4
**Figure S4**	Rate of CENTA hydrolysis by NDM-1 in assay buffer with $ZnSO_4$	S4
**Figure S5**	Rate of CENTA hydrolysis by NDM-1 in $ZnSO_4$ and EDTA	S4
**Figure S6**	Mass Spectra of the PDTC complexes collected in ESI (-) mode	S5
**Table S1**	PDTC complex and PDTC MIC data*	S6
**Table S2**	Metal salt MIC data*	S6
**Table S3**	Meropenem MIC data with metal salts*	S6

***** Organisms tested*: E. coli 2692, E. coli BAA-2452, and K. pneumonieae BAA-2146*

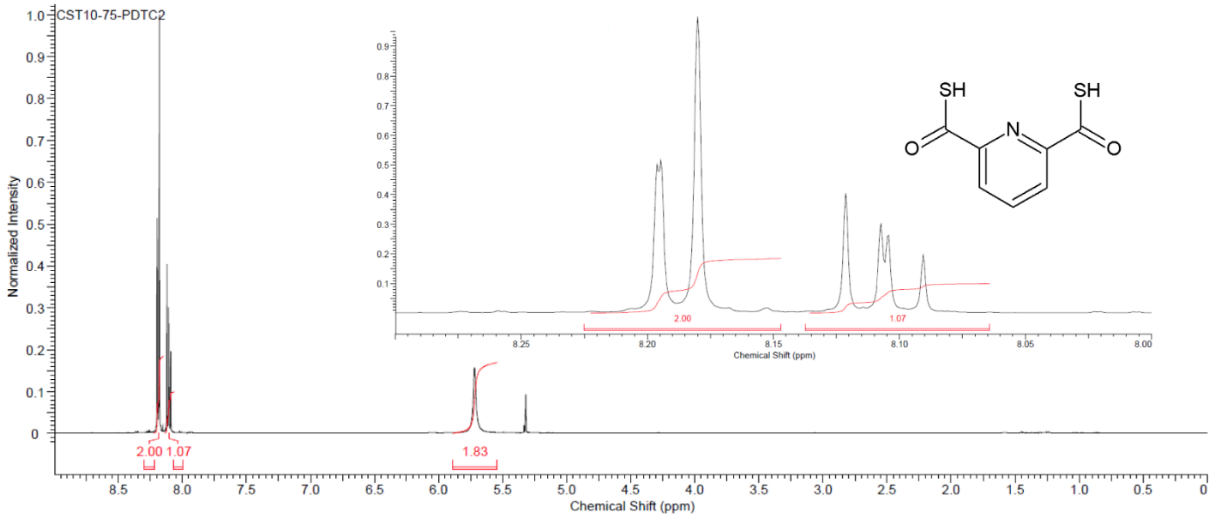

Figure S1. ^{1}H NMR spectrum and integration of synthesized PDTC. Inset expands the region between 8 and 8.3 ppm to highlight the second order coupling pattern – pattern identified as AB_2.

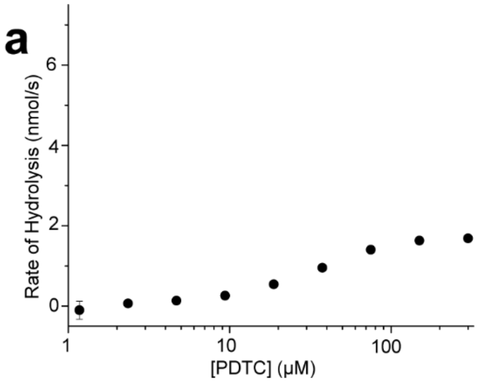

Figure S2. Rate of CENTA hydrolysis in the presence of PDTC with no NDM-1.

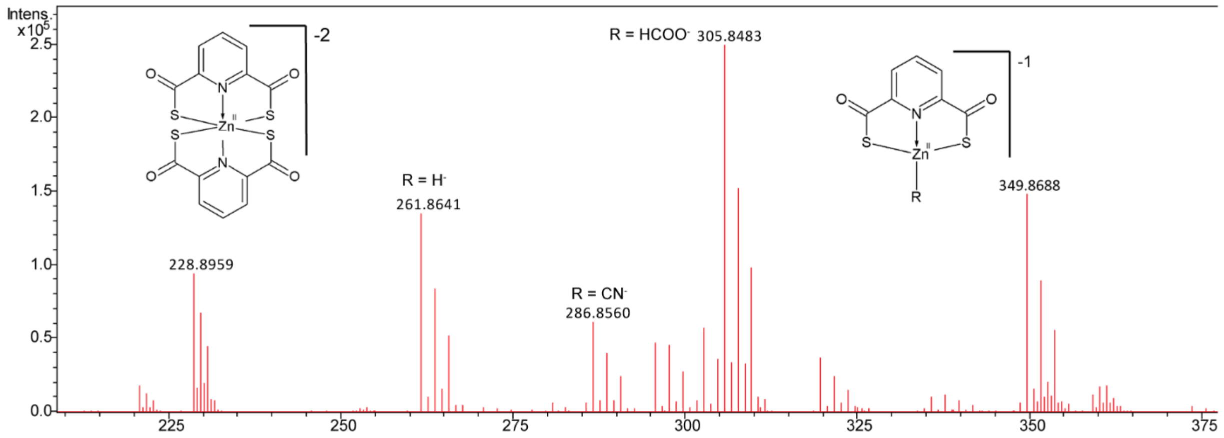

Figure S3. Mass spectra acquired for PDTC in the presence of $ZnSO_4$, revealing a 2:1 $PDTC_2$-Zn complex for 228.8959 m/z $[M-2H]^{-2}$. Several 1:1 PDTC-Zn complexes were identified where able.

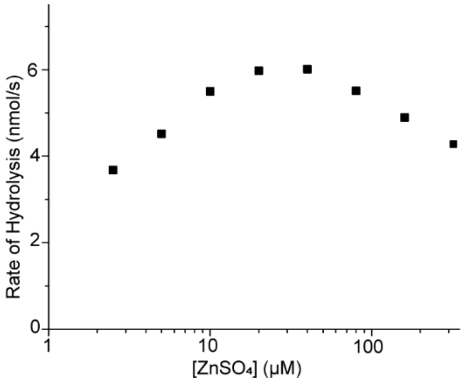

Figure S4. Rate of CENTA hydrolysis by NDM-1 at different concentrations of $ZnSO_4$ in the assay buffer. Optimal rate of hydrolysis takes place between 20 and 40 μM.

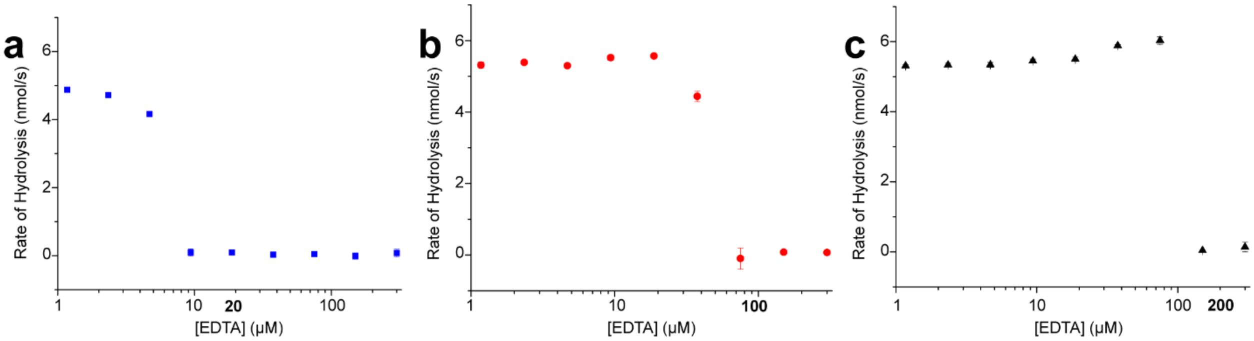

Figure S5. Initial rates of CENTA hydrolysis by NDM-1 vs. EDTA concentration with (**a**) 10 μM $ZnSO_4$, (**b**) 50 μM $ZnSO_4$, (**c**) 100 μM $ZnSO_4$ supplementing the reaction buffer. EDTA eliminates in a 1:1 equivalency with the amount of $ZnSO_4$ incorporated, as compared with PDTC, which reduced activity at 2 equiv. of $ZnSO_4$ (denoted by bold type on x axis).

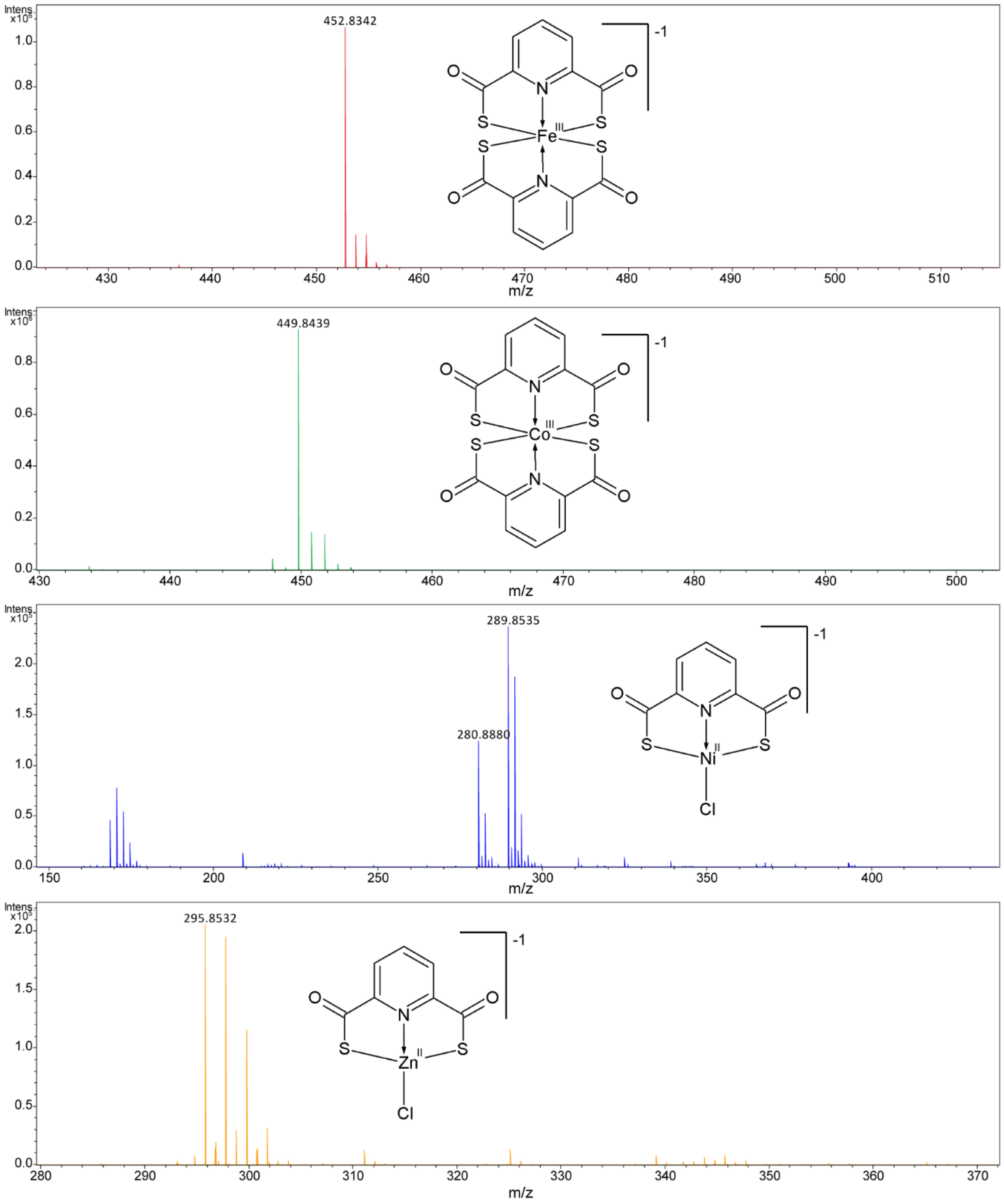

Figure S6. Mass Spectra of the PDTC complexes collected in ESI (-) mode.

Table S1. PDTC complex and PDTC MIC data. MICs shown are an average of 4 replicates.

	Observed MIC (mM)		
	E. coli 2692	*E. coli* BAA-2452	*K. pneumonieae* BAA-2146
$PDTC_2$-Fe	>1.6	>1.6	1.6
$PDTC_2$-Co	>1.6	>1.6	1.6
PDTC-Ni	>1.6	>1.6	1.6
PDTC-Zn	0.2	0.2	0.4
PDTC (no metal)	0.4	0.4	0.4

Table S2. Metal salt MIC data. MICs shown are an average of 4 replicates.

	Observed MIC (mM)		
	E. coli 2692	*E. coli* BAA-2452	*K. pneumonieae* BAA-2146
$ZnCl_2$	> 1.6	> 1.6	1.6
$FeCl_3$	> 1.6	> 1.6	> 1.6
$NiCl_2$	> 1.6	> 1.6	> 1.6
$CoCl_2$	1.6	> 1.6	1.6

Table S3. Meropenem MIC data with metal salts. MICs shown are an average of 4 replicates.

	Observed Meropenem MIC (μg/mL)		
	E. coli 2692	*E. coli* BAA-2452	*K. pneumonieae* BAA-2146
(no salt)	64	16	128
$ZnCl_2$	64	32	128
$FeCl_3$	64	8	128
$NiCl_2$	64	32	128
$CoCl_2$	64	10	64

Metal salts were screened at a constant concentration of 400 μM, equivalent to 0.25 x MIC or the highest concentration tested for Table S2
